# From Berries to Capsules: Technological and Quality Aspects of Juneberry Formulations

**DOI:** 10.3390/ph18121841

**Published:** 2025-12-02

**Authors:** Lauryna Pudžiuvelytė, Agnė Mačiulskaitė

**Affiliations:** 1 Department of Drug Technology and Social Pharmacy, Lithuanian University of Health Sciences, LT-50161 Kaunas, Lithuania; 2 Institute of Pharmaceutical Technologies, Lithuanian University of Health Sciences, LT-50161 Kaunas, Lithuania

**Keywords:** Juneberry, Saskatoon, *Amelanchier alnifolia*, berries, capsules, antioxidant activity, freeze-drying, polyphenols, powders

## Abstract

**Background:** *Amelanchier alnifolia* (Juneberry) is a phenolic-rich species with potential for pharmaceutical applications. This study aimed to optimize ultrasound-assisted extraction (UAE) conditions for producing ethanolic extracts from differently processed Juneberry berries collected in Lithuania and to develop solid oral dosage forms based on the obtained extracts. **Methods:** Extracts were prepared using varying ethanol concentrations, temperatures, and extraction times from dried, frozen, and freeze-dried berries. Total phenolic content (TPC) and total flavonoid content (TFC) were determined spectrophotometrically. Antioxidant activity was evaluated by DPPH and ABTS assays. Phenolic profiles were quantified by high-performance liquid chromatography (HPLC), identifying five major compounds. Extracts were converted into powders using lactose monohydrate, microcrystalline cellulose, or magnesium aluminum metasilicate as carriers. Hard capsules were manufactured and evaluated according to European Pharmacopoeia (Ph. Eur.) requirements, including mass uniformity, moisture content, and disintegration time. **Results:** Freeze-dried berries yielded the highest TPC, TFC, and antioxidant activity across all extraction conditions. The most efficient extraction parameters for freeze-dried berries were identified as 50% ethanol, 50–55 °C, and 30 min. HPLC analysis confirmed the presence of chlorogenic and neochlorogenic acids, rutin, hyperoside, and isoquercitrin. Among the powdered systems, lactose monohydrate demonstrated favorable flowability and moisture characteristics. **Conclusions:** Freeze-dried Juneberry berries are a suitable raw material for producing phenolic-rich extracts with strong antioxidant activity. Lactose-based powder blends showed the best technological performance and were successfully formulated into hard capsules. These findings support the potential of Juneberry extracts for incorporation into standardized pharmaceutical dosage forms and provide a basis for future formulation and bioavailability studies.

## 1. Introduction

**Figure 1:**
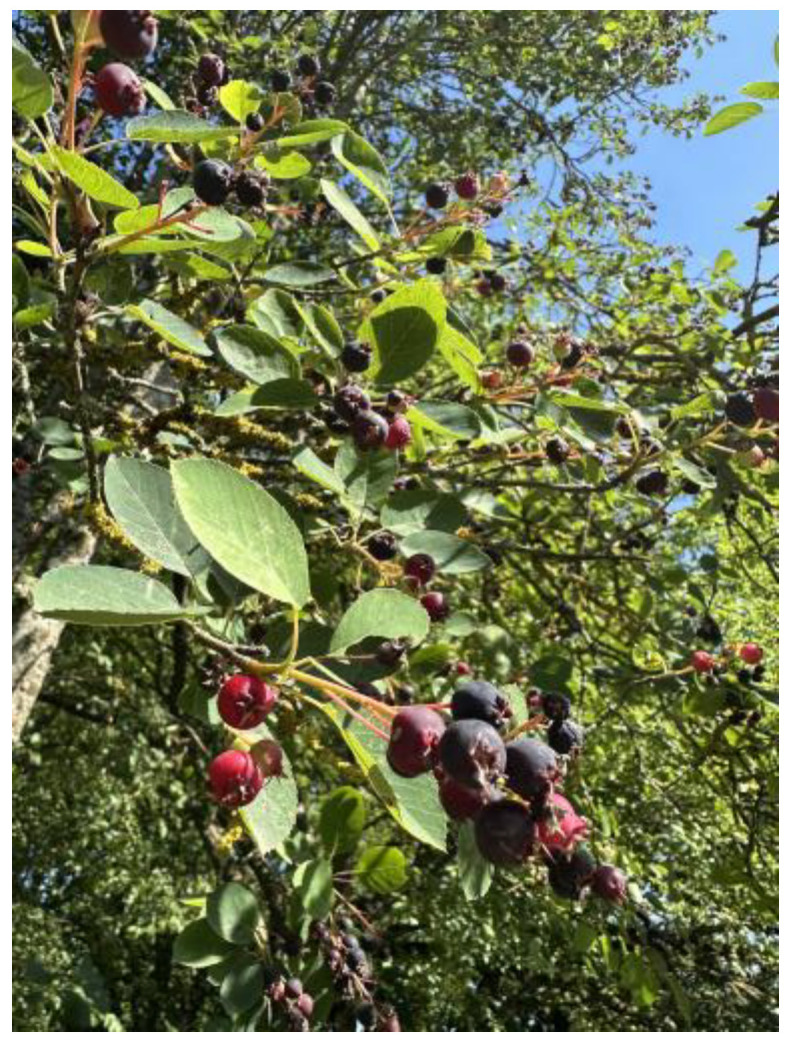
Juneberry berries. Photos by authors.

Juneberry, also known as Saskatoon (*Amelanchier alnifolia* Nutt.) is a shrub belonging to the rose family (*Rosaceae*). The medicinal plant material is the fruit—small, dark purple to black berries (Figure 1) that contain flavonoids, phenolic acids, triterpenoids, and anthocyanins [1,2]. Since it can grow in various soil types, does not require special cultivation conditions (e.g., high temperature or humidity), and is resistant to frost, it can be successfully grown in Lithuania. It is a relatively understudied plant species, which, due to the compounds it accumulates, has demonstrated antiviral activity against intestinal coronavirus and the ability to modulate lipid metabolism and energy expenditure [3,4]. It is believed that, through its capacity to neutralize free radicals, it can delay the onset of diseases associated with oxidative stress [5]. Studies show that it exhibits anticancer, anti-inflammatory, antifungal, antiatherosclerotic, hypoglycemic, antihypertensive, antiallergic, antiradical, neuroprotective, and vasoprotective effects [2,6,7,8]. In addition, due to their radical-scavenging properties, phenolic compounds are valuable in the food industry, as they can extend the shelf life of products by mitigating lipid oxidation and preserving quality parameters such as color, taste, smell, and texture [5].

Although the phytochemical composition of Juneberry has been partially described in previous studies [1,2,5], there is still limited research exploring the technological translation of its bioactive-rich extracts into standardized pharmaceutical dosage forms. Furthermore, there is a distinct lack of experimental studies investigating *A. alnifolia* berries collected and processed under the climatic and soil conditions specific to Lithuania. Such regional variations may significantly influence the phytochemical composition and antioxidant potential of the plant material [9], underscoring the importance of conducting localized experimental assessments.

Juneberry has been traditionally used in North America by Indigenous peoples for both nutritional and medicinal purposes, primarily as an anti-inflammatory and energy-restoring food source [10]. Beyond its ethnobotanical value, *A. alnifolia* has attracted increasing scientific interest due to its high content of anthocyanins, flavonols, and phenolic acids, which are associated with strong antioxidant and health-promoting properties [2,8,11]. In Canada and the northern United States, Juneberries are cultivated commercially and utilized in a wide range of food and nutraceutical products, including juices, jams, yogurts, functional beverages, and dietary supplements [2,12]. In recent years, their extracts have also been incorporated into cosmetic formulations for skin protection against oxidative stress [13] and evaluated for potential use in encapsulated delivery systems in pharmaceutical and nutraceutical industries [14]. However, while such applications are growing internationally, there remains a scarcity of data on the phytochemical and technological properties of Lithuanian-grown Juneberries, making this research both regionally significant and scientifically relevant.

In order to preserve as much of the biologically active material as possible, it is essential to consider the factors that influence the yield of phenolic compounds. The most commonly used extraction methods include maceration, supercritical-fluid extraction, ultrasonic extraction, and microwave-assisted extraction. Once the appropriate extraction conditions are determined, the maximum yield of bioactive compounds can be achieved [15,16].

Therefore, the rationale of this study is based on the hypothesis that optimizing the ultrasonic-assisted extraction parameters (solvent concentration, temperature, and extraction time) from differently processed Juneberry berries (frozen, dried, freeze-dried) can significantly influence phenolic compounds, flavonoids, and antioxidant activity, and consequently the quality of pharmaceutical formulations derived from these extracts.

Once the extracts are obtained, they can be incorporated into powders using liquid–solid phase technology to preserve the physicochemical and biological properties of the bioactive compounds. Thanks to this technology, the resulting powder exhibits good flowability, non-stickiness, and enhanced solubility [17,18]. The prepared powder mixtures can then be used for the production of hard capsules—one of the oldest and most convenient oral dosage forms. Capsules effectively mask unpleasant tastes and odors, protect the active ingredient from environmental degradation (e.g., light, oxygen, microbial contamination), and allow accurate dosing of liquids or solids [19,20].

Given the broad pharmacological potential of Juneberry and its favorable growth characteristics in Lithuania, as well as the scarcity of experimental research on locally grown berry material, the development of standardized dosage forms containing its extracts is of both scientific and practical importance. Therefore, the aim of this work was to produce liquid ethanol extracts from Juneberry berries and to determine the effect of extraction time, temperature, and solvent concentration on the content of phenolic compounds and flavonoids, as well as on antioxidant activity, and to evaluate the technological quality of hard capsules formulated from these extracts.

## 2. Results and Discussion

### 2.1. The Influence of Extraction Conditions on the Qualitative and Quantitative Assessment of Phenolic Compounds in Extracts Prepared from Juneberry Berries

The aim of this study was to determine the optimal extraction time, temperature, and ethanol concentration for producing liquid ethanol extracts from Juneberry berries using UAE. Extraction conditions are summarized in Table 1.

In order to use the extract with the highest concentration of extracted compounds in production, antioxidant activity was assessed, and the total content of phenolic compounds and flavonoids was determined.

### 2.2. Determination of the Total Phenolic Content in Extracts Prepared from Juneberry Berries Depending on Extraction Conditions

**Figure 2:**
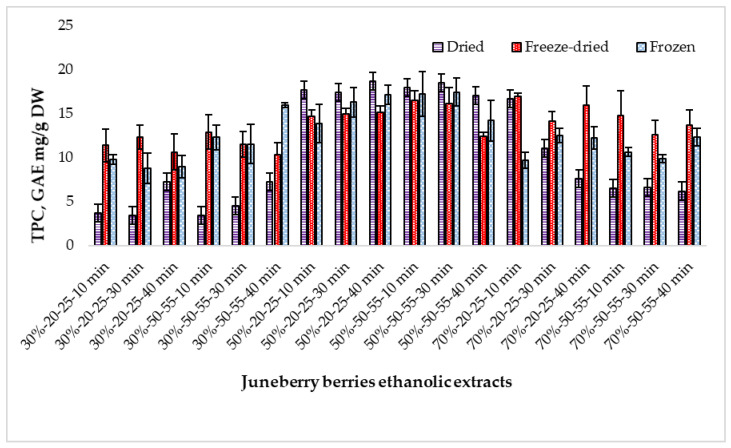
Total phenolic content (TPC) in extracts prepared from dried, freeze-dried, and frozen Juneberry berries obtained under different ethanol concentrations, extraction temperatures, and extraction times. First number (30%, 50%, and 70%)—ethanol concentration; middle numbers (20–25 and 50–55)—extraction temperature range in °C; last number (10 min, 30 min, and 40 min)—extraction duration.

The chemical composition of Juneberry processed under different conditions was evaluated through the determination of total phenolic content (TPC), flavonoids, and antioxidant activity. Figure 2 shows the distribution of TPC.

Across all raw material types, a consistent trend was observed: freeze-dried berries yielded the highest phenolic levels, followed by frozen and dried material. TPC ranged from 3.37 to 18.69 GAE mg/g DW, with freeze-dried berries showing the greatest content (18.69 ± 0.18 GAE mg/g DW) and dried berries the lowest (3.37 ± 0.13 GAE mg/g DW), with statistically significant differences between freeze-dried and dried samples (*p* < 0.001) (Supplementary Material Table S1).

Extraction temperature (20–25 °C vs. 50–55 °C) produced only minor fluctuations within each raw material group, and no statistically significant effect on TPC was detected (*p* > 0.05). Similarly, extraction time (10, 30, and 40 min) resulted in small numerical differences that were not statistically significant (*p* > 0.05). These findings indicate that neither temperature nor time was a major determinant of extraction efficiency within the tested ranges. (Supplementary Material Tables S3 and S4).

In contrast, ethanol concentration had a pronounced and statistically supported influence on phenolic yield. For dried berries, TPC increased from 4.92 ± 1.87 GAE mg/g DW (30%) to 17.87 ± 0.63 GAE mg/g DW (50%), before decreasing at 70% ethanol (9.11 ± 4.12 GAE mg/g DW). A similar pattern was observed for freeze-dried berries, where 50% ethanol resulted in the highest TPC (14.95 ± 1.45 GAE mg/g DW), significantly exceeding both 30% and 70% ethanol (*p* < 0.001). Therefore, ethanol concentration—particularly at 50%—was identified as the primary factor affecting phenolic extraction, regardless of the berry processing method. (Supplementary Material Table S2).

The obtained data were compared with results from other studies analyzing phenolic compounds in *Amelanchier* species. Didur et al. [15] reported that the TPC in *A. alnifolia* fruits reached 194.27 ± 7.14 GAE mg/100 g fresh weight (FW) in the pulp and 511.47 ± 13.91 GAE mg/100 g FW in the peel. When expressed per gram of fresh weight, these values (approximately 1.9–5.1 mg/g) are notably lower than those obtained in this study (3.37–18.69 mg/g). Similarly, Koron and Mikulic-Petkovsek [9] reported phenolic concentrations of ~298 mg/100 mL in *A. lamarckii* juice. Lachowicz et al. (2019) examined the influence of drying methods on *A. alnifolia* and observed TPC values ranging from 3.21 ± 0.22 mg/g DW (microwave-vacuum drying) to 6.84 ± 0.18 mg/g DW (freeze-drying) [2].

Such differences may result from variations in solvent polarity, extraction technique, and the expression of results on a dry-weight versus fresh-weight basis. Nevertheless, all studies confirm that drying method and solvent concentration play a crucial role in determining the phenolic yield from Juneberry berries.

### 2.3. Determination of the Total Flavonoid Content in Extracts Prepared from Juneberry Berries Depending on Extraction Conditions

**Figure 3:**
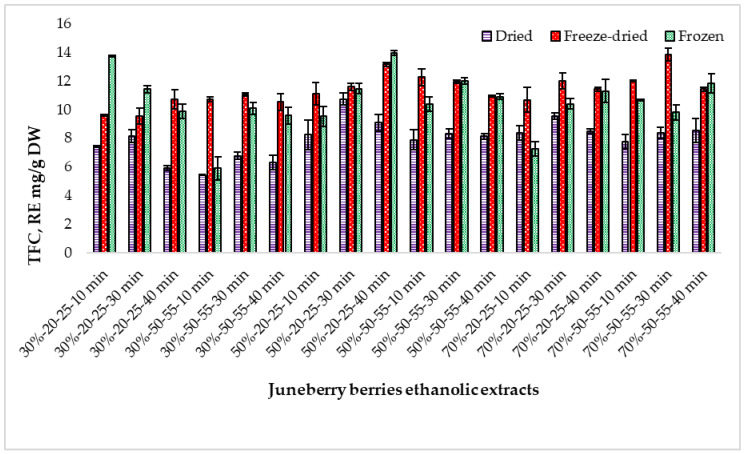
Total flavonoid content (TFC) in extracts prepared from dried, freeze-dried, and frozen Juneberry berries obtained under different ethanol concentrations, extraction temperatures, and extraction times. First number (30%, 50%, and 70%)—ethanol concentration; middle numbers (20–25 and 50–55)—extraction temperature range in °C; last number (10 min, 30 min, and 40 min)—extraction duration.

TFC showed a consistent pattern across all Juneberry processing methods, with freeze-dried berries yielding the highest values, followed by frozen and dried material. TFC ranged from 5.45 to 13.71 mg RE/g DW, and all three raw material types differed significantly from each other (*p* < 0.05) (Figure 3).

Ethanol concentration had a notable effect on flavonoid extraction. For all berry types, 50% and 70% ethanol produced significantly higher TFC than 30% ethanol, while no meaningful difference occurred between 50% and 70% (see Supplementary Material for statistics). Freeze-dried extracts extracted with 50–70% ethanol reached 11.83–11.88 mg RE/g DW, compared with 10.36 mg RE/g DW at 30%. A similar trend was observed in frozen and dried samples, confirming that medium–high ethanol concentrations improve flavonoid yield. (Supplementary Material Tables S5 and S6).

Extraction temperature (20–25 °C vs. 50–55 °C) resulted in only minor numerical changes, none of which were statistically significant. Likewise, extraction time (10, 30, and 40 min) caused minimal variation in TFC for freeze-dried and frozen berries, with only a single significant comparison between 10 and 30 min in dried samples. Overall, neither temperature nor time meaningfully influenced flavonoid extraction within the tested ranges. (Supplementary Material Tables S7 and S8).

Taken together, these findings demonstrate that raw material type and ethanol concentration are the primary determinants of TFC, with freeze-dried berries and 50–70% ethanol consistently providing the highest flavonoid levels. Freeze-dried berry extracts consistently exhibited the highest TFC, while dried berry extracts showed the lowest values. This trend aligns with earlier studies reporting superior polyphenol retention in freeze-dried *Amelanchier* berries [2,9]. The higher absolute values obtained in the present study likely result from the expression of TFC on a dry-weight basis and the use of concentrated ultrasonic extracts.

The UAE has emerged over the last decade as a robust, green technology for maximizing recovery of bioactive phenolics and flavonoids from Vaccinium matrices, including bilberry (*V. myrtillus*) and American cranberry (V. macrocarpon). Studies on bilberry pomace have shown that extending extraction from 15 to 45 min in combination with ultrasound can approximately double TPC and increase TFC by more than twofold, even in simple aqueous systems, highlighting the strong mass-transfer enhancement provided by acoustic cavitation [21]. Optimized UAE of bilberry leaves using hydroethanolic solvents (typically 30–50% ethanol, *v*/*v*) and short sonication times (≈5–15 min) has yielded markedly high TPC values, exceeding 200 GAE mg/g DW, while preserving antioxidant activity [22,23]. Similarly, work on wild bilberry juice by-products and cranberry press residues demonstrates that hydroethanolic mixtures of 40–70% ethanol outperform absolute ethanol, with TPC in cranberry residues reaching roughly 1.6–3.0 g/100 g under 10–40 min of UAE and extraction kinetics generally plateauing after about 15 min [24,25]. In contrast, conventional techniques such as long maceration, Soxhlet extraction, or non-sonicated shaking consistently deliver lower phenolic yields under comparable solvent conditions [24,25]. Collectively, these findings indicate that UAE with 40–70% ethanol–water mixtures, moderate temperatures (approximately 40–60 °C), and extraction times in the range of 10–30 min offers an efficient and scalable strategy to obtain phenolic- and flavonoid-rich bilberry and cranberry extracts suitable for incorporation into pharmaceutical dosage forms and functional formulations [21,22,23,24,25,26].

### 2.4. Determination of Antioxidant Activity Using the ABTS Method

**Figure 4:**
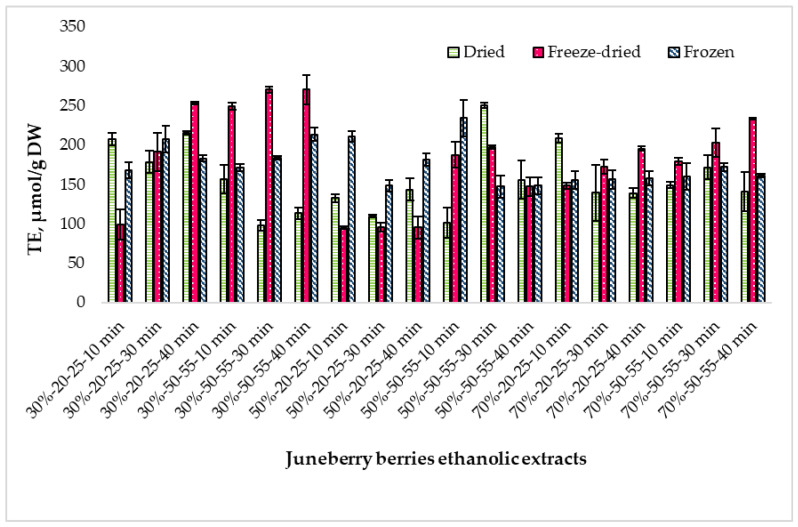
Trolox equivalent (TE) antioxidant capacity by ABTS method of extracts prepared from dried, freeze-dried, and frozen Juneberry berries and obtained under different ethanol concentrations, extraction temperatures, and extraction times. First number (30%, 50%, and 70%)—ethanol concentration; middle numbers (20–25 and 50–55)—extraction temperature range in °C; last number (10 min, 30 min, and 40 min)—extraction duration.

Figure 4 shows the changes in ABTS free radical scavenging activity in extracts prepared from differently processed Juneberry berries.

ABTS radical scavenging activity differed depending on the berry processing method. Freeze-dried extracts showed the highest values (182.24 ± 0.43 µmol TE/g DW), followed by frozen material (175.41 ± 0.44 µmol TE/g DW) and dried berries (156.06 ± 0.47 µmol TE/g DW). Tukey HSD indicated a statistically significant difference only between freeze-dried and dried samples (*p* < 0.01), confirming that raw material processing has a substantial effect on ABTS activity (Supplementary Material Table S9).

Ethanol concentration also influenced antioxidant capacity. Across all material types, extracts prepared with 50% and 70% ethanol showed significantly higher ABTS activity than those made with 30% ethanol (*p* < 0.0001 and *p* < 0.05, respectively), while no significant difference was observed between 50% and 70% ethanol (*p* > 0.05). This effect was most pronounced in freeze-dried berries, where ABTS activity reached up to 222.15 ± 9.72 µmol TE/g DW at 30% ethanol and 188.41 ± 17.99 µmol TE/g DW at 70%. (Supplementary Material Table S10).

Extraction temperature had a clear and statistically supported impact. Extracts obtained at 50–55 °C exhibited significantly higher ABTS values compared with those extracted at 20–25 °C across most raw material types (*p* < 0.01). This suggests that moderate heating improves the extraction of ABTS-reactive compounds. (Supplementary Material Table S11).

Extraction time (10, 30, 40 min) caused only minor fluctuations in ABTS activity, and Tukey HSD confirmed no statistically significant differences among the time points for any raw material (*p* > 0.05). Thus, prolonging sonication beyond 10 min did not increase ABTS capacity within the tested range. (Supplementary Material Table S12).

Overall, ABTS antioxidant activity was primarily determined by raw material type, ethanol concentration, and extraction temperature, while extraction time showed no significant effect. Freeze-dried berry extracts consistently demonstrated the strongest antioxidant potential.

### 2.5. Determination of Antioxidant Activity Using the DPPH Method

**Figure 5:**
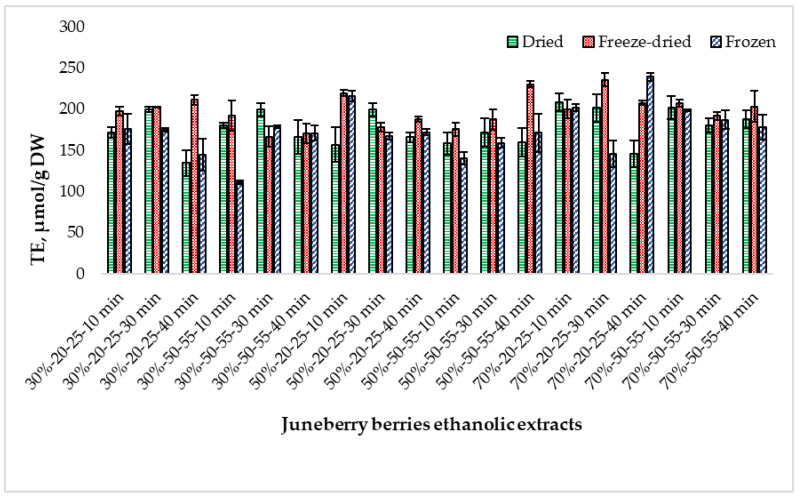
Trolox equivalent (TE) antioxidant capacity by the DPPH method of extracts prepared from dried, freeze-dried, and frozen Juneberry berries and obtained under different ethanol concentrations, extraction temperatures, and extraction times. First number (30%, 50%, and 70%)—ethanol concentration; middle numbers (20–25 and 50–55)—extraction temperature range in °C; last number (10 min, 30 min, and 40 min)—extraction duration.

DPPH radical scavenging activity ranged from 134.28 to 235.70 µmol TE/g DW across all extracts (Figure 5). Freeze-dried berries demonstrated the highest antioxidant activity (235.70 ± 2.36 µmol TE/g DW), followed by frozen material, while dried berries showed the lowest activity (134.28 ± 2.69 µmol TE/g DW). Tukey HSD confirmed that freeze-dried extracts had significantly higher DPPH activity than both frozen and dried samples (*p* < 0.001), whereas dried and frozen extracts did not differ significantly (Supplementary Material Table S13).

Ethanol concentration strongly influenced DPPH results. For all berry types, extracts prepared with 70% ethanol exhibited significantly higher activity compared with those extracted with 30% or 50% ethanol (*p* < 0.001). No significant difference was observed between 30% and 50% ethanol (*p* > 0.05). In freeze-dried berries, DPPH activity increased from 188.55 ± 13.20 µmol TE/g DW (30%) to 207.30 ± 10.37 µmol TE/g DW (70%), illustrating the enhancing effect of higher ethanol content on the extraction of DPPH-reactive compounds. (Supplementary Material Table S14).

Extraction temperature showed a moderate effect. Across most raw material types, extracts obtained at 50–55 °C displayed slightly higher DPPH activity compared with those extracted at 20–25 °C, and this difference reached statistical significance (*p* < 0.05). This suggests that elevated temperatures may improve mass transfer or solubility of specific antioxidant constituents. (Supplementary Material Table S15).

Extraction time (10, 30, 40 min) produced only numerical fluctuations in DPPH activity, and Tukey HSD indicated no statistically significant differences among time intervals for any berry type (*p* > 0.05) (Supplementary Material Table S16). Thus, prolonging extraction beyond 10 min did not meaningfully enhance DPPH antioxidant capacity. Overall, DPPH activity was primarily influenced by raw material processing and ethanol concentration, with freeze-dried berries and 70% ethanol consistently providing the highest radical-scavenging activity. Extraction temperature had a secondary but significant effect, while extraction time did not contribute significantly within the studied range.

Other researchers have also investigated the antioxidant properties of *A. alnifolia* and related species under comparable conditions. Researchers [8] examined the effect of different drying methods (freeze-drying, microwave-vacuum, hot-air, and vacuum drying) on the antioxidant retention of Saskatoon berries. They reported that freeze-dried berries exhibited the highest DPPH scavenging activity, confirming that lyophilization preserves antioxidant compounds most effectively. These results align closely with the present study, where the antioxidant activity reached 77% in freeze-dried berries compared with 59% in dried and 36% in frozen samples. Li et al. [27] studied phenolic composition and antioxidant activity of *A. alnifolia* pomace extracts and found that the values of methanol extracts ranged from 54.2 to 119.4 µmol TE/g DW of pomace and ethanol extracts from 53.4 to 103.1 µmol TE/g DW of pomace. The ORAC value of the extract was significantly (*p* < 0.05) affected by the extraction solvent, and a similar trend was shown in TPC and flavonoid content. De Souza et al. [5] examined the phenolic composition and antioxidant properties of dried *Amelanchier* berry pomace and reported that the antioxidant activity strongly depended on solvent polarity. Extracts obtained with 40% ethanol showed the highest DPPH (16.1 ± 0.5 1/IC_50_) and ABTS (197.6 ± 4.4 mM TEAC/100 mg FW) activities among the tested solvent fractions, indicating that medium-polarity mixtures (30–50% ethanol) are optimal for extracting phenolic antioxidants from Saskatoon berry matrices.

Correlation analysis revealed distinct relationships between total flavonoid content (TFC) and antioxidant activity parameters. A weak but significant positive correlation was observed between TFC and DPPH radical-scavenging activity (r = 0.293, *p* = 0.03), indicating that higher flavonoid levels were associated with slightly increased DPPH capacity. No significant correlation was found between TFC and ABTS activity (r = 0.108, *p* = 0.462), suggesting that ABTS-reactive compounds may not be directly linked to flavonoid concentration. A moderate positive correlation was detected between TFC and total phenolic content (TPC) (r = 0.4625, *p* = 0.0004), confirming that flavonoids contribute substantially to the total phenolic pool in Juneberry extracts. Together, these findings indicate that the antioxidant response is driven by multiple compound classes, with flavonoids contributing more strongly to DPPH activity and overall phenolic content than to ABTS reactivity. (Supplementary Material Figures S6–S8).

### 2.6. High–Performance Liquid Chromatography Method for Quantitative Determination

Five compounds were identified using high–performance liquid chromatography: neochlorogenic acid, chlorogenic acid, rutin, hyperoside, and isoquercitrin (Supplementary Material Figures S1–S3). The content of phenolic compounds (µg/g DW) in Juneberry berry extracts under different extraction conditions is shown in Table 2.

The highest total content of identified phenolic compounds was found in liquid extracts prepared from freeze-dried Juneberry berries, reaching 159.618 µg/g DW, while the lowest total amount was detected in extracts from dried berries (60.700 µg/g DW). Among all identified compounds, chlorogenic acid was the dominant component. Its concentration was the highest in extracts prepared from frozen berries (103.219 µg/g DW), which is consistent with previous studies on *A. alnifolia*, where chlorogenic acid has been described as the major hydroxycinnamic acid [1,2,15]. Similar findings were obtained by other researchers [1,2], with rutin, hyperoside, and quercetin derivatives being among the main flavonoids identified in Juneberry berries and extracts.

Other phenolic compounds detected in the samples included neochlorogenic acid, rutin, hyperoside, isoquercitrin, and quercetin. The highest concentration of neochlorogenic acid occurred in extracts prepared from frozen berries (17.762 µg/g DW), while the lowest—extracts prepared from dried berries (2.723 µg/g DW). Rutin content was greatest in extracts obtained from freeze-dried berries (22.530 µg/g DW) and lowest in extracts prepared from frozen berries (7.886 µg/g DW). The highest amount of hyperoside was also found in extracts prepared from freeze-dried berries (2.500 µg/g DW), with the lowest level detected in extracts prepared from frozen berries (1.247 µg/g DW). Isoquercitrin showed the highest concentration in extracts prepared from freeze-dried berries (39.205 µg/g DW) and the lowest—extracts obtained from dried berries (24.669 µg/g DW).

### 2.7. Production of Powder with Liquid Extract in Liquid–Solid Phase

Based on a comprehensive analysis of TPC, TFC, ABTS, and DPPH data, the most effective extraction conditions for obtaining high-value bioactive compounds from Juneberry were identified as the use of freeze-dried raw material, extraction with a 50% ethanol–water mixture, and sonication at 50–55 °C for approximately 30 min. These conditions consistently yielded high levels of phenolic compounds and flavonoids, together with strong antioxidant activity. Among all evaluated variables, raw material type and ethanol concentration were the most influential factors, whereas extraction time showed no significant effect within the tested range.

These extraction parameters were selected following a comparative evaluation of all combinations of ethanol concentration (30, 50, and 70%), temperature (20–25 °C and 50–55 °C), and extraction time (10, 30, and 40 min). Extracts prepared from freeze-dried berries consistently demonstrated the highest TPC, TFC and antioxidant activity, and within this group, the combination of 50% ethanol and moderate heating (50–55 °C) provided the most favorable balance between phenolic yield and antioxidant capacity. Increasing the temperature from 20–25 °C to 50–55 °C in extracts prepared from freeze-dried berries notably enhanced ABTS activity, while changes in TPC and TFC remained minimal, indicating that moderate heating improves extraction efficiency without causing degradation of phenolic compounds.

Extraction times were selected to reflect the typical kinetic profile of UAE. Although extending sonication from 10 to 30 min increased TFC to some extent, it had no meaningful effect on TPC or antioxidant activity according to DPPH and ABTS assays. Therefore, 30 min were considered an optimal compromise between extraction efficiency and practical applicability.

Although the maximum values of individual parameters (TPC, TFC, DPPH, ABTS) did not occur under identical conditions, optimal extraction was determined based on a holistic assessment. While 70% ethanol produced the highest DPPH activity, it resulted in lower phenolic yields and was less suitable for downstream drying and liquid–solid processing. Conversely, 30% ethanol produced higher ABTS values only under specific conditions but did not lead to comparable TPC or TFC levels. Taken together, freeze-dried material extracted with 50% ethanol at 50–55 °C for 30 min provided the most balanced outcome—high phenolic content, strong and thermally stable antioxidant activity, and favorable technological properties of the resulting extract.

In addition to phytochemical advantages, the use of 50% ethanol also offers technological, economic, and environmental benefits. Lower solvent concentration reduces the consumption of organic solvents and the energy required for their removal, making the process more cost-efficient and environmentally sustainable. Extracts obtained with 50% ethanol are easier to evaporate, concentrate, or dry, which is especially advantageous for liquid–solid systems and other dosage forms, as the lower alcohol content accelerates solvent removal and reduces thermal stress on thermolabile compounds. Thus, the selected extraction conditions are not only chemically and biologically optimal but also well-suited for scalable pharmaceutical and nutraceutical manufacturing.

Using liquid–solid phase technology, three carriers were selected: lactose monohydrate, microcrystalline cellulose, and magnesium aluminum metasilicate. The excipients selected in this study—lactose monohydrate, microcrystalline cellulose, and magnesium aluminum metasilicate—are well established and widely used in pharmaceutical formulation science due to their safety, biocompatibility, and functional versatility. Lactose monohydrate is a pharmaceutically accepted diluent recognized for its non-toxicity, chemical inertness, and ability to improve flow and compressibility of powders [28]. Microcrystalline cellulose is a GRAS-status excipient used as a binder and disintegrant; it is chemically stable, non-reactive with phenolic and flavonoid compounds, and compatible with a wide range of active substances [29]. Magnesium aluminum metasilicate (Neusilin) is an inert, amorphous adsorbent that enhances powder flow, increases surface area, and stabilizes moisture-sensitive extracts [30]. These excipients are regarded as non-toxic, non-irritant, and safe for oral administration, and are commonly used in solid dosage forms such as tablets, capsules, and granules. Their physicochemical compatibility with both hydrophilic and lipophilic compounds ensures formulation stability and reproducibility. For further experiments, an extract was prepared from freeze-dried berries, with 50% ethanol, extraction time 30 min, at 50–55 °C.

**Figure 6:**
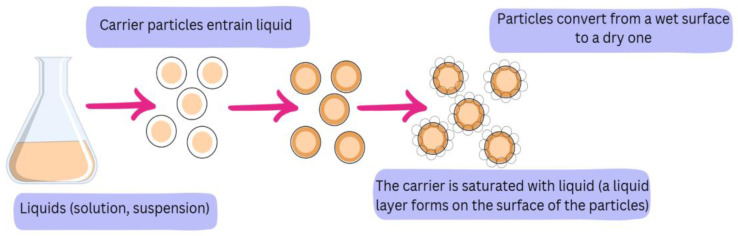
Representation of liquid–solid technique.

The active ingredient used was a liquid ethanol extract of freeze-dried Juneberry berries. Figure 6 shows the liquid–solid schematic technique representation.

An analysis of the appearance of the powders was performed. The fine, shiny powder of lactose monohydrate with Juneberry extract stood out with its pink color and a strong characteristic odor.

Microcrystalline cellulose blended with the extract exhibited a light pink coloration and a faint characteristic odor, whereas the magnesium aluminum metasilicate mixture showed no visible reddish hue, retained its inherent white appearance, and demonstrated a neutral odor.

### 2.8. Determination of Moisture Content in Powder

The moisture content was determined using a Kern MLS 50–3 HA 160 moisture analyzer (Kern & Sohn GmbH, Balingen, Germany) at 105 °C. Moisture was measured both in powders without the active ingredient and in powders containing the active ingredient. A reduction in moisture content was observed in all samples. One measurement was performed for each powder, and the results are presented in Table 3.

The initial moisture content of microcrystalline cellulose was 4.67%, which decreased to 3.81% after the addition of the ethanol extract of freeze-dried Juneberry berries. The moisture content of lactose monohydrate decreased from 2.57% to 1.36% following extract incorporation. Magnesium aluminum metasilicate exhibited an initial moisture content of 10.33%, while the corresponding powder containing Juneberry extract showed a reduced value of 8.99%.

Based on these data, all powders met the Ph. Eur. requirements, which specify that the moisture content must not exceed 13% unless otherwise stated. The results demonstrated that the addition of Juneberry extract improved the moisture characteristics of the powders by reducing their residual moisture content.

### 2.9. Determination of Powder Flowability and Angle of Repose

The Carr’s index and Hausner ratio were used to evaluate the flowability of the powders. The results obtained for density, flowability, and angle of repose are presented in Table 4. A lower Carr’s index indicated better powder flowability, while a lower Hausner ratio likewise reflected improved flow characteristics. A Hausner ratio greater than 1.34 was considered indicative of poor flowability.

Based on these parameters, lactose monohydrate powder containing freeze-dried Juneberry extract demonstrated the best flowability (classified as average flowability), whereas magnesium aluminum metasilicate powder containing berry extract showed the poorest (extremely poor) flowability. A statistically significant difference in flow time was observed between Neusilin^®^ US2 and lactose monohydrate powders, as well as a significant difference in the angle of repose between lactose monohydrate with Juneberry extract and Neusilin^®^ US2 (*p* < 0.05). Significant differences were also found in tapped density between microcrystalline cellulose and microcrystalline cellulose with Juneberry extract (*p* < 0.05), and between lactose monohydrate and lactose monohydrate with berry extract (*p* < 0.05).

The results showed that the lactose monohydrate powder containing Juneberry extract exhibited the most favorable angle of repose (27° ± 2), which falls within the optimal range of 25–30°. All powders demonstrated acceptable flowability, as the expected flowability for suitable powders is 4–5 g/s. The findings indicated that the addition of liquid Juneberry extract significantly improved the technological properties of the powders, including flow time, flowability, angle of repose, tapped and bulk density, as well as the Hausner ratio and Carr’s index. Because the lactose monohydrate–Juneberry extract mixture exhibited the most favorable characteristics, it was selected for the production of hard capsules. Based on the results of antioxidant activity, phenolic content, and flavonoid content, the freeze-dried berry extract obtained using 50% ethanol, a 30 min extraction time, and a temperature of 50–55 °C was chosen for encapsulation.

Overall, the results demonstrated that the selected carriers were technologically appropriate and chemically compatible with the freeze-dried Juneberry extract. No undesired physicochemical interactions were observed, and the resulting powder mixtures exhibited suitable flowability and homogeneity for encapsulation. Future studies will include differential scanning calorimetry (DSC) and Fourier-transform infrared spectroscopy (FTIR) analyses to further confirm the absence of chemical interactions between the extract and the excipients.

### 2.10. Determination of Capsule Weight Uniformity

Capsule weight uniformity was determined to ensure consistent dosing. The results are presented in Table 5.

According to the Ph. Eur., the permissible deviation in capsule weight is ±7.5%. Twenty capsules from each batch were weighed, and the average weight was calculated to assess compliance with these requirements. The lowest capsule mass was observed in formulations containing magnesium aluminum metasilicate (Neusilin^®^ US2) (0.124 ± 0.009 g), while the highest mass was recorded for capsules containing lactose monohydrate (0.347 ± 0.007 g). A statistically significant difference was found between capsules with Neusilin^®^ US2 and those with microcrystalline cellulose (*p* < 0.05). Overall, the results demonstrated that all capsule formulations met the Ph. Eur. criteria, as none deviated from the mean capsule mass by more than 7.5%.

### 2.11. Determination of Capsule Disintegration Time

The aim of this study was to evaluate the disintegration time of capsules containing Juneberry berry extract formulated with different excipients. The capsules were stored at room temperature prior to testing. According to the Ph. Eur., capsules were required to disintegrate within 30 min. The results are presented in Table 6. The longest disintegration time was observed in capsules containing magnesium aluminum metasilicate (Neusilin^®^ US2) (4.5 ± 1 min), whereas the shortest disintegration time was recorded for capsules formulated with lactose monohydrate (3.5 ± 1 min).

Based on the results obtained, it was concluded that the capsules formulated with these excipients met the Ph. Eur. disintegration time requirements, as their disintegration times were below 30 min (1800 s).

## 3. Materials and Methods

### 3.1. A. alnifolia Extracts Preparation

**Figure 7:**
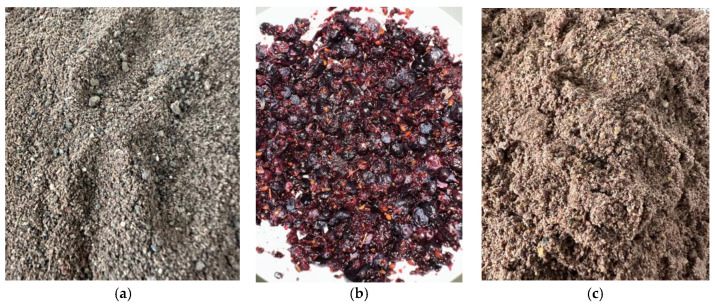
(**a**) berries, dried in a dryer and crushed; (**b**) frozen berries crushed in a mortar; (**c**) berries, freeze-dried and crushed. Photos by authors.

The exact amount of crushed Juneberry berries (1.0 g) (Figure 7) was weighed using analytical scales. The material was mixed with 20 mL of ethanol (30%, 50%, or 70%) and placed in an ultrasound-assisted extraction bath (Bandelin electronic GmbH & Co.KG, Berlin, Germany) at the required temperature (20–25 °C or 50–55 °C) for the designated extraction time (10, 30, or 40 min. All extractions were performed using berries from the same batch, and all analytical results were expressed on a dry-weight (DW) basis to ensure comparability between samples with different initial moisture contents. After extraction, the samples were filtered through 100-µm pore membrane filters.

### 3.2. The Total Amount of Phenolic Compounds

The total amount of phenolic compounds was determined using UV/VIS 1800 Shimadzu spectrophotometer (Shimadzu, Tokyo, Japan) applying a modified Folin–Ciocalteu method. In total, 0.2 mL of extract was diluted with 4 mL of purified water and then mixed. Then, 5 mL of Folin–Ciocalteu reagent was added to the diluted extract, and after 5 min, 4 mL of 7.5% sodium carbonate solution added. The reference solution was prepared in the same way as the test solution, but 0.2 mL of extractant was used instead of the extract. The prepared solutions are stored at room temperature, protected from light. After one hour, the absorbance of the solutions was measured with a spectrophotometer at a wavelength of 765 nm. Gallic acid, a phenolic compound, was used to prepare a reference standard curve. A gallic acid stock solution (1 mg/mL) was used to prepare working concentrations (0.1–1 mg/mL), which were obtained by serial dilution using Milli-Q water. Calibration curve y = 0.2018x − 0.0039; R^2^ = 0.9984. (Supplementary Material Figure S4).

### 3.3. Determination of Total Flavonoid Content

The TFC was determined by spectrophotometric analysis. In total, 1 mL of the test extract was added; 2 mL of 96% ethanol (*v*/*v*), 0.1 mL of 30% aqueous acetic acid solution, 0.3 mL of 10% aqueous aluminum (III) hydroxide solution, and 0.4 mL of 5% aqueous hexamethylenetetramine solution were added to a 25 mL measuring flask and mixed thoroughly. The solutions were diluted with purified water to the mark, kept at room temperature for 30 min in the dark. The light absorption was measured with a spectrophotometer at a wavelength of 407 nm. Rutin, a phenolic compound, was used to prepare a reference standard curve. Rutin stock solution (1 mg/mL) was used to prepare working concentrations (0.1–1 mg/mL), which were obtained by serial dilution using Milli-Q water. Calibration curve y = 1.2294x + 0.1661; R^2^ = 0.9851. (Supplementary Material Figure S5).

### 3.4. Determination of Antioxidant Activity Using the DPPH Method

Free radical scavenging method. A working solution was prepared by dissolving 0.0017 g of DPPH reagent in 100 mL of 96.3% (*v*/*v*) ethanol. In total, 20 μL of the test extract and 3 mL of the working DPPH solution were added to a test tube. The solution was mixed and kept in the dark for 30 min at room temperature. The reference solution was ethanol. After 30 min, the absorbance of the test solutions was measured with a spectrophotometer at a wavelength of 517 nm. Calibration curve y = 0.000012x + 0.0177685; R^2^ = 0.9941.

### 3.5. Determination of Antioxidant Activity Using the ABTS Method

Radical–cation binding method. In total, 0.0548 g of weighed ABTS powder was placed in a dark glass bottle and dissolved in 50 mL of purified water using ultrasonic waves. In total, 0.0095 g of potassium peroxodisulfate was added to the resulting solution. We closed the bottle, mixed the solution, and left it in the dark for 12–16 h. After 12–16 h, the stock ABTS solution was diluted with purified water until an absorption value of 0.700 was obtained at a wavelength of 734 nm. The reference solution was purified water. To perform the test, we took 3 mL of the working ABTS solution, added 20 μL of the test extract, mixed it well, and kept it in the dark for 1 h. After one hour, the absorbance was measured with a spectrophotometer at a wavelength of 734 nm. Calibration curve y = 0.00003x − 0.00340; R^2^ = 0.9718.

### 3.6. Quantitative Evaluation of Juneberry Berries Extracts Using HPLC

HPLC analysis was conducted to evaluate the content of phenolic compounds in extracts prepared from Juneberry berries. A Waters 2695 chromatograph (Waters, Milford, MA, USA) with a Waters 996 photodiode array detector was used for the study. A YMC–Pack ODS–A column (250 × 4.6 mm) with a sorbent particle size of 5 μm (YMC Europe GmbH, Dinslaken, Germany) was used to determine quercetin glycosides and phenolic acids. The mobile phase consisted of two substances: acetonitrile (*v*/*v*) and 2% acetic acid (*v*/*v*). The flow rate of the mobile phase was 1.0 mL/min, and the analysis time was 24 min. The temperature was maintained at 25 °C. The injection volume of the test sample was 10 μL. A Waters 2695 chromatograph with a Waters 996 photodiode array detector and AGE 5C18 (250 × 4.6 mm) column and precolumn (Advanced Chromatography Technologies, Aberdeen, Scotland) was used to determine chlorogenic acids. More information about HPLC analysis parameters is placed in the Supplementary Material section.

### 3.7. Production of Powder Containing Ethanol Extract of Juneberry Berries in a Liquid–Solid Phase System

**Figure 8:**
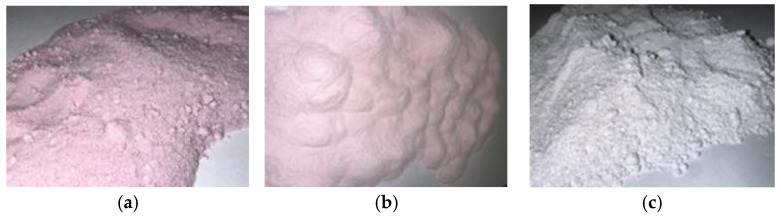
(**a**) Lactose monohydrate powder with Juneberry extract; (**b**) Microcrystalline cellulose powder with Juneberry extract; (**c**) Neusilin^®^ US2 powder with Juneberry extract. Photos by authors.

In total, 50 g of the carrier and 40.0 g of liquid Juneberry extract were mixed in a mortar. The prepared mixture was then placed in a drying oven at 60 °C for 1 h. This step was repeated until all of the extract had been used. After the entire amount of extract was incorporated, the resulting mixture was dried at 40 °C for 24 h. The dried powders are shown in Figure 8.

The dried powders were transferred into dark glass bottles and stored at room temperature in the absence of light until further analysis.

### 3.8. Moisture Content of Powder

The moisture content of the powder was determined using a moisture analyzer. One gram of the powder was dried at 105 °C until a constant mass was achieved. The moisture content was measured three times, and the average value was calculated. The result was expressed as the percentage of mass loss during drying. According to the requirements, the moisture content was not permitted to exceed 13%.

### 3.9. Determination of Powder Flowability and Angle of Repose

The flowability test was performed to determine the ability of the powder to flow vertically from a container under the influence of gravity (Ph. Eur. 2.9.36). For this test, 30 g of powder was weighed and poured into a dry funnel equipped with a slide valve. The apparatus was switched on, and the powder was shaken in a vibrating device for 20 s. Immediately afterward, the valve at the bottom of the funnel was opened, and the stopwatch was started simultaneously. The time required for the entire powder portion to flow out of the funnel was recorded. The test was repeated three times.

### 3.10. Determination of Tapped Density

Tapped density was determined by mechanically compacting the powder. An accurately weighed amount of powder (to 0.01 g precision) was transferred into a measuring cylinder. The appropriate parameters were selected, and the measurement was initiated while the change in powder volume was monitored. Powder volume was recorded after 10, 490, 750, and 1250 taps. The test was completed when the powder volume in the cylinder no longer changed. After the procedure, the device provided the following data: tapped density, bulk density, Carr’s index, and Hausner’s ratio.

### 3.11. Capsule Technology

**Figure 9:**
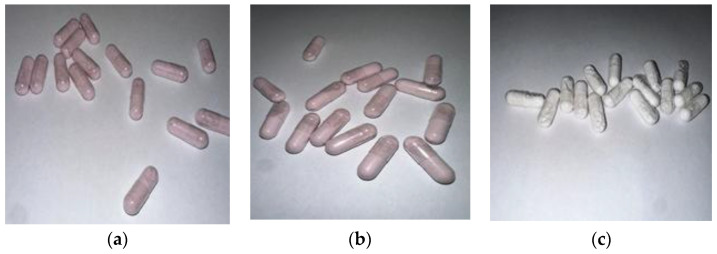
(**a**) Capsules with lactose monohydrate and Juneberry extract; (**b**) Capsules with microcrystalline cellulose and Juneberry extract; (**c**) Capsules with Neusilin and Juneberry extract. Photos by authors.

Capsules were manufactured using a hand-operated encapsulation device equipped with plastic trays containing 15 slots for size 1 capsules. The capsules were separated, and the bodies were placed into the designated slots. The powder blend was evenly distributed over the tray, allowing the capsule bodies to be filled by gravity and gentle leveling. The caps were then aligned and secured by applying light pressure with the upper plate of the device. After sealing, the filled capsules were released from the tray and collected for further evaluation (Figure 9).

The manufactured capsules were transferred into dark glass bottles and stored at room temperature in the absence of light until further analysis.

### 3.12. Determination of Mass Uniformity

The capsule mass uniformity test was performed according to the method described in Ph. Eur. 2.9.5. For this test, 20 random capsules were taken and weighed, after which the average capsule mass was calculated. Each capsule was then opened, and its entire contents were emptied and weighed separately. The empty capsule shell was also weighed. The mass of the capsule contents was determined by subtracting the mass of the empty shell from the mass of the intact capsule. This procedure was repeated for all remaining capsules. According to the Ph. Eur. requirements, no more than two of the tested capsules were allowed to deviate by more than 10% from the average capsule mass when the average mass was below 300 mg, or by more than 7.5% when the average mass exceeded 300 mg. Additionally, none of the capsules were permitted to exceed twice these deviation limits.

### 3.13. Determination of Capsule Disintegration Time

The capsule disintegration time was determined using a magnetic stirrer. A laboratory glass containing 50 mL of purified water and a magnetic bar was placed on the magnetic stirrer and heated to 37 ± 0.5 °C at 700 rpm. A capsule was then placed into the glass, and the start time of the test was recorded. When the capsule fully disintegrated, the end time was noted. The procedure was performed with six capsules. If at least one of the six capsules failed to disintegrate within the time specified in the Ph. Eur., an additional twelve capsules were tested. In this case, at least sixteen out of the eighteen capsules were required to disintegrate within the specified time.

### 3.14. Data Analysis

Statistical analysis of the research data was performed using Microsoft 365 Excel (Microsoft, Redmond, WA, USA) and SPPS Statistics 29 (IBM, Armonk, NY, USA). Three technical replicates of each sample were measured. The arithmetic mean and standard deviations of the results obtained were calculated. Statistical significance was determined using one–way analysis of variance (ANOVA) with Tukey Post Hoc (HSD) criterion. Differences were considered statistically significant when *p* < 0.05.

## 4. Conclusions

This study demonstrated that the technological properties and phytochemical composition of Juneberry berry extracts strongly depend on the processing method of the raw material and the extraction parameters. Among the evaluated matrices, freeze-dried berries consistently produced extracts with the highest total phenolic content, flavonoid levels, and antioxidant activity (ABTS, DPPH), confirming that freeze-drying is the most suitable preprocessing technique for preserving bioactive compounds.

UAE using 50% ethanol at 50–55 °C for 30 min was identified as the optimal condition for freeze-dried berries. HPLC analysis revealed that chlorogenic and neochlorogenic acids, rutin, hyperoside, and isoquercitrin were the predominant constituents across all extracts, with the highest overall phenolic yield achieved in freeze-dried berry extracts.

The liquid–solid conversion of the optimal extract produced powders with acceptable technological characteristics. Lactose monohydrate demonstrated the most favorable flowability, density parameters, and moisture content after extract incorporation, making it the most suitable carrier for encapsulation. All hard capsules met the Ph. Eur. requirements for mass uniformity and disintegration time, confirming the technological feasibility of formulating Juneberry extract into a solid oral dosage form.

Overall, the results indicate that freeze-dried Juneberry berries are a rich and stable source of phenolic antioxidants suitable for pharmaceutical applications. Future studies should explore additional dosage forms, assess bioavailability, stability, and investigate the potential of fresh berries to further refine formulation strategies.

## Figures and Tables

**Table 1:** The experimental extraction conditions for liquid extracts preparation from Juneberry frozen, freeze-dried, and dried berries.

Factor A	Factor B	Factor C
Extraction Time (min)	Temperature (°C)	Ethanol Concentration (%)
10, 30, 40	20–25 50–55	30, 50, 70

**Table 2:** Content of phenolic compounds of extracts prepared with frozen, freeze-dried, and dried Juneberry berries.

Compound	Class	Retention Time (min)	Amount of Compounds in Liquid Extracts Prepared from Juneberry Berries, µg/g DW		
			Frozen	Freeze-Dried	Dried
Neochlorogenic acid	Hydroxycinnamic acid	9.326	17.762	14.277	2.723
Chlorogenic acid	Hydroxycinnamic acid	11.854	103.219	81.104	15.410
Rutin	Flavonol glycoside	22.870	7.886	22.530	15.352
Hyperoside	Flavonol glycoside	23.465	1.247	2.500	1.739
Isoquercitrin	Flavonol glycoside	24.317	26.107	39.205	24.669

**Table 3:** Moisture content of the powder (n = 3).

Powders	Factor
	Dry Matter Content, %
Microcrystalline cellulose	4.67 ± 0.08
Lactose monohydrate	2.57 ± 0.11
Magnesium aluminum metasilicate (Neusilin^®^ US2)	10.33 ± 0.15
Microcrystalline cellulose and *A. alnifolia* liquid extract	3.81 ± 0.13
Lactose monohydrate and *A. alnifolia* liquid extract	1.36 ± 0.12
Magnesium aluminum metasilicate (Neusilin^®^ US2) and *A. alnifolia* liquid extract	8.99 ± 0.09

**Table 4:** Technological properties of the powders (n = 3).

Powders	Factor A	Factor B	Factor C	Factor D	Factor E	Factor F	Factor G
	Disintegration Time (s)	Flowability (g/s)	Cone Angle (˚)	Tapped Density (g/cm^3^)	Bulk Density (g/cm^3^)	Carr’s Index (%)	Hausner Ratio
Microcrystalline cellulose	46.7 ± 14.6	1.182 ± 0.001	47 ± 3	0.458 ± 0.006	0.351 ± 0.001	23.393 ± 1.010	1.305 ± 0.018
Lactose monohydrate	46.7 ± 19.9	0.574 ± 0.001	47 ± 1	0.833 ± 0.001	0.546 ± 0.023	34.47 ± 2.767	1.528 ± 0.064
Neusilin^®^ US2	51.7 ± 2.6	0.287 ± 0.001	52 ± 2	0.156 ± 0.005	0.169 ± 0.003	30.937 ± 1.071	1.448 ± 0.023
Microcrystalline cellulose and *A. alnifolia* liquid extract	36.7 ± 4.9	1.765 ± 0.001	37 ± 1	0.522 ± 0.008	0.39 ± 0.009	25.280 ± 2.840	1.339 ± 0.050
Lactose monohydrate and *A. alnifolia* liquid extract	26.7 ± 5.9	1.097 ± 0.001	27 ± 2	0.69 ± 0.001	0.566 ± 0.009	17.907 ± 1.328	1.218 ± 0.020
Neusilin^®^ US2 and *A. alnifolia* liquid extract	35.7 ± 5.4	0.347 ± 0.001	36 ± 1	0.164 ± 0.008	0.101 ± 0.001	38.333 ± 3.215	1.626 ± 0.086

**Table 5:** Uniformity of capsule weight (n = 3).

Capsule Composition	Factor
	Capsule Mass Uniformity, g
Lactose monohydrate and *A. alnifolia* liquid extract	0.347 ± 0.007
Microcrystalline cellulose and *A. alnifolia* liquid extract	0.256 ± 0.009
Neusilin^®^ US2 and *A. alnifolia* liquid extract	0.124 ± 0.009

**Table 6:** Capsule disintegration time (n = 3).

Capsule Composition	Factor
	Capsule Disintegration Time (min)
Lactose monohydrate and *A. alnifolia* liquid extract	4 ± 0.75
Microcrystalline cellulose and *A. alnifolia* liquid extract	3.5 ± 1
Neusilin^®^ US2 and *A. alnifolia* liquid extract	4.5 ± 1

## Data Availability

The original contributions presented in this study are included in the article/Supplementary Material. Further inquiries can be directed to the corresponding author.

## Supplementary Materials

The following supporting information can be downloaded at: https://www.mdpi.com/article/10.3390/ph18121841/s1, Figure S1: Representative HPLC-PDA chromatograms (λ = 360 nm) of phenolics in extracts of Juneberry dried berries; Figure S2: Representative HPLC-PDA chromatograms (λ = 360 nm) of phenolics in extracts of Juneberry frozen berries; Figure S3: Representative HPLC-PDA chromatograms (λ = 360 nm) of phenolics in extracts of Juneberry freeze-dried berries; Figure S4: Calibration curve for gallic acid in the spectrophotometric assay; Figure S5: Calibration curve for rutin in the spectrophotometric assay; Figure S6: Correlation Between Total Flavonoid Content (TFC) and DPPH Radical-Scavenging Activity; Figure S7: Correlation Between Total Flavonoid Content (TFC) and ABTS Radical-Scavenging Activity; Figure S8: Correlation Between Total Flavonoid Content (TFC) and Total Phenolic Content (TPC); Table S1: ANOVA Tukey HSD pairwise comparisons for the effect of raw material type on TPC; Table S2: ANOVA Tukey HSD pairwise comparisons for the effect of ethanol concentration on TPC; Table S3: ANOVA Tukey HSD pairwise comparisons for the effect of temperature on TPC; Table S4: ANOVA Tukey HSD pairwise comparisons for the effect of extraction time on TPC; Table S5: ANOVA Tukey HSD pairwise comparisons for the effect of raw material type on TFC; Table S6: ANOVA Tukey HSD pairwise comparisons for the effect of ethanol concentration on TFC; Table S7: ANOVA Tukey HSD pairwise comparisons for the effect of temperature on TFC; Table S8: ANOVA Tukey HSD pairwise comparisons for the effect of extraction time on TFC; Table S9: ANOVA Tukey HSD pairwise comparisons for the effect of raw material type on antioxidative activity by ABTS method; Table S10: ANOVA Tukey HSD pairwise comparisons for the effect of ethanol concentration on antioxidative activity by ABTS method; Table S11: ANOVA Tukey HSD pairwise comparisons of the effect of temperature on antioxidative activity by ABTS method; Table S12: ANOVA Tukey HSD pairwise comparisons for the effect of time on antioxidative activity by ABTS method; Table S13: ANOVA Tukey HSD pairwise comparisons for the effect of raw material type on antioxidative activity by DPPH method; Table S14: ANOVA Tukey HSD pairwise comparisons for the effect of ethanol concentration on antioxidative activity by DPPH method; Table S15: ANOVA Tukey HSD pairwise comparisons of the effect of temperature on antioxidative activity by DPPH method; Table S16: ANOVA Tukey HSD pairwise comparisons for the effect of time on antioxidative activity by DPPH method.

## Abbreviations

The following abbreviations are used in this manuscript:

ABTS	2,2′-azino-bis(3-ethylbenzothiazoline-6-sulfonic acid)
DPPH	2,2-diphenyl-1-picrylhydrazyl
DW	Dry weight
DSC	Differential scanning calorimetry
Ph. Eur.	European Pharmacopoeia
FTIR	Fourier-transform infrared spectroscopy
GAE	Gallic acid equivalents
HPLC	High-performance liquid chromatography
RE	Rutin equivalents
TPC	Total phenolic content
TFC	Total flavonoid content
UAE	Ultrasound-assisted extraction
