# Supplementary material for: From Berries to Capsules: Technological and Quality Aspects of Juneberry Formulations
Source: Pharmaceuticals (Basel). 2025 Dec 2;18(12):1841. doi: 10.3390/ph18121841 (PMC12736035; doi:10.3390/ph18121841)
Supplement: Supplementary file 1 [file pharmaceuticals-18-01841-s001.zip › pharmaceuticals-3984849-supplementary.pdf]

**Supplementary Material**

*Quantitative evaluation of Juneberry berries extracts using HPLC*

Mobile phase materials: a mixture of phosphoric acid, acetonitrile, and purified water (1:19:80 v/v/v) and a mixture of phosphoric acid, methanol, and acetonitrile (1:40:59 v/v/v). Analysis time – 31 min. Mobile phase flow rate – 1.2 mL/min. Test sample injection volume – 10 μL. UV detector wavelength – 360 nm. For quantitative analysis, a calibration curves were obtained by injecting known concentrations (0.5–100 mg/mL) of different standard compounds. All calibration curves demonstrated excellent linearity, with correlation coefficients ($R^2$) ranging from 0.9982 to 0.9997. The limit of detection (LOD) and limit of quantification (LOQ) were calculated using the standard deviation of the response and the slope of the calibration curve (LOD = 3.3σ/S, LOQ = 10σ/S). LOD values ranged from 0.12 to 0.35 μg/mL, and LOQ values ranged from 0.40 to 1.15 μg/mL, depending on the analyte. All compounds demonstrated %RSD values below 3%, indicating good repeatability. Retention time variability was below 1%. Accuracy was evaluated by recovery testing at 80%, 100%, and 120% spiking levels, yielding values between 96.2% and 103.5%, confirming good recovery and absence of matrix effects. These results confirm that the method is sensitive, linear, precise, and suitable for the quantitative evaluation of phenolic compounds in Juneberry extracts. Concentrations were determined and summed using Epower 3 chromatography software (Waters Corporation, Milford, USA).

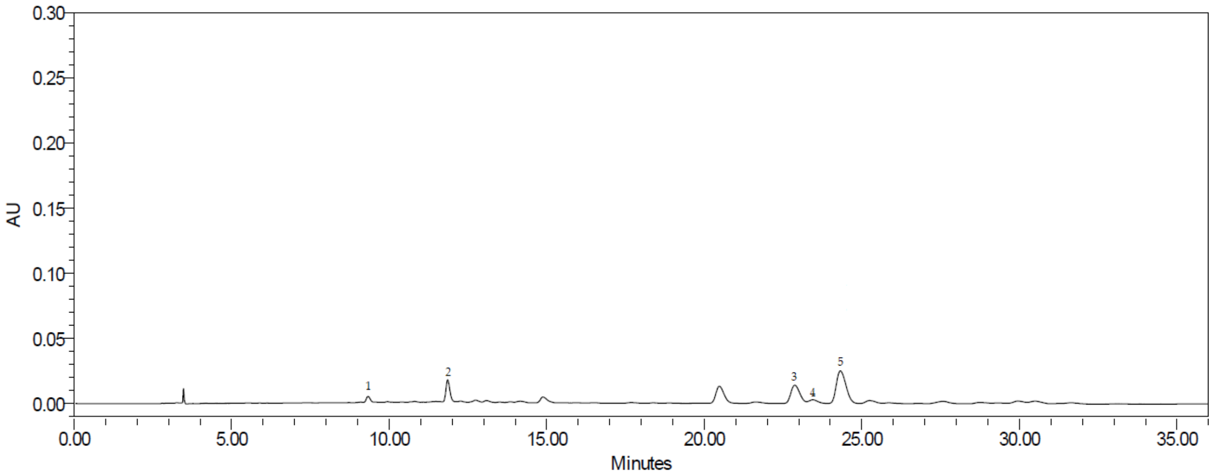


**Figure S1.** Representative HPLC-PDA chromatograms (λ = 360 nm) of phenolics in extracts of Juneberry dried berries. Peak assignments: 1 – neochlorogenic acid; 2 – chlorogenic acid; 3 – rutin; 4 – hyperoside; 5 isoquercitrin.

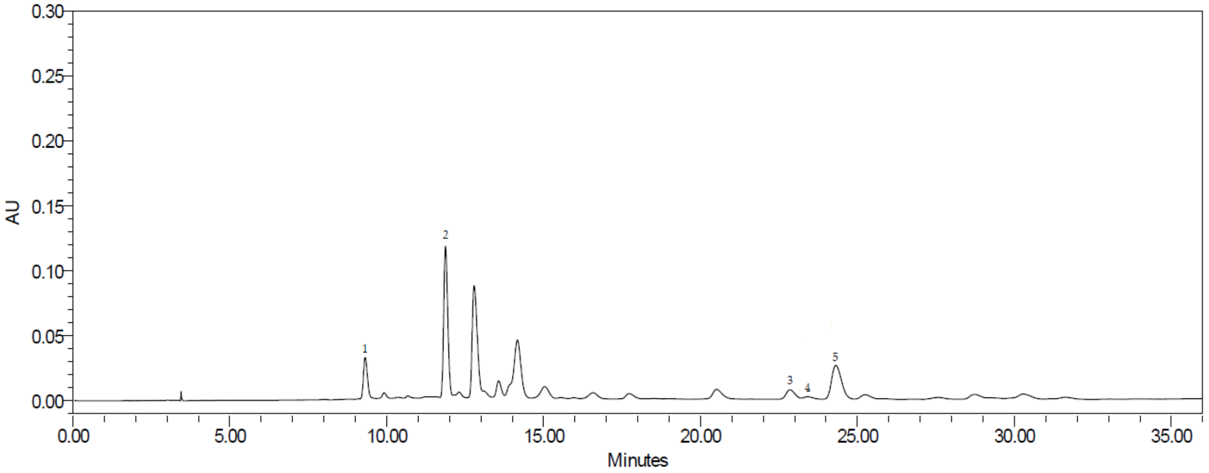


**Figure S2.** Representative HPLC-PDA chromatograms ($\lambda$ = 360 nm) of phenolics in extracts of Juneberry frozen berries. Peak assignments: 1 – neochlorogenic acid; 2 – chlorogenic acid; 3 – rutin; 4 – hyperoside; 5 isoquercitrin.

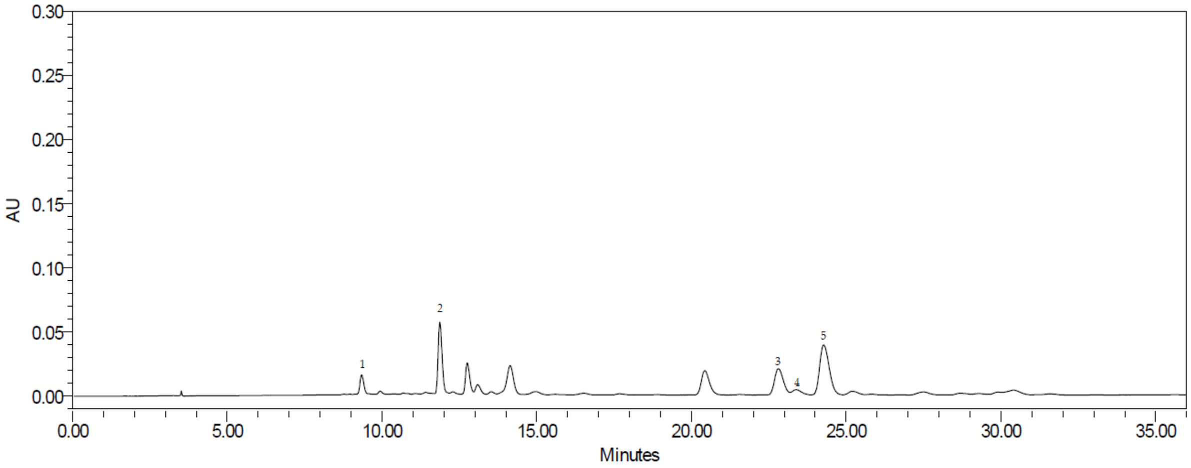


**Figure S3.** Representative HPLC-PDA chromatograms ($\lambda$ = 360 nm) of phenolics in extracts of Juneberry freeze-dried berries. Peak assignments: 1 – neochlorogenic acid; 2 – chlorogenic acid; 3 – rutin; 4 – hyperoside; 5  isoquercitrin.

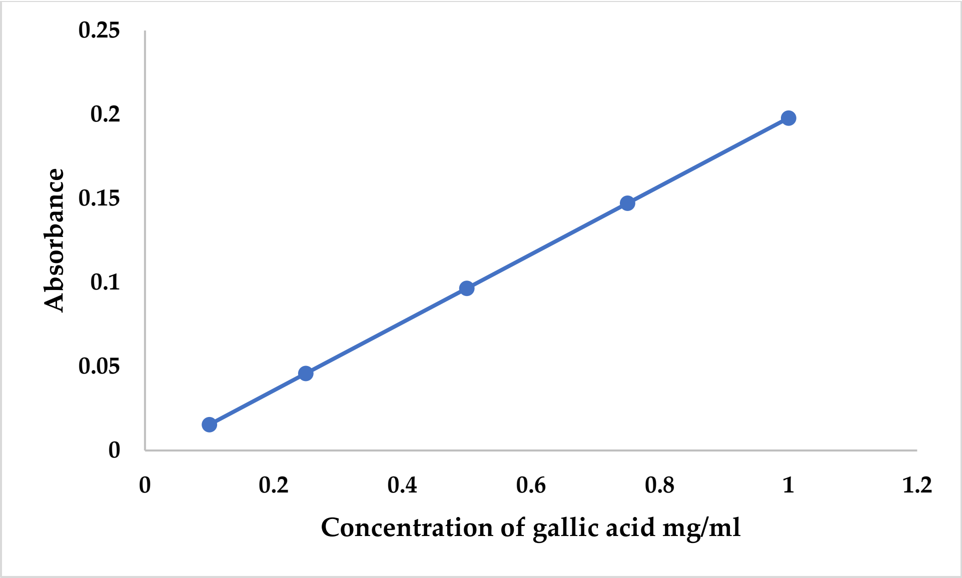


**Figure S4.** Calibration curve for gallic acid in the spectrophotometric assay.

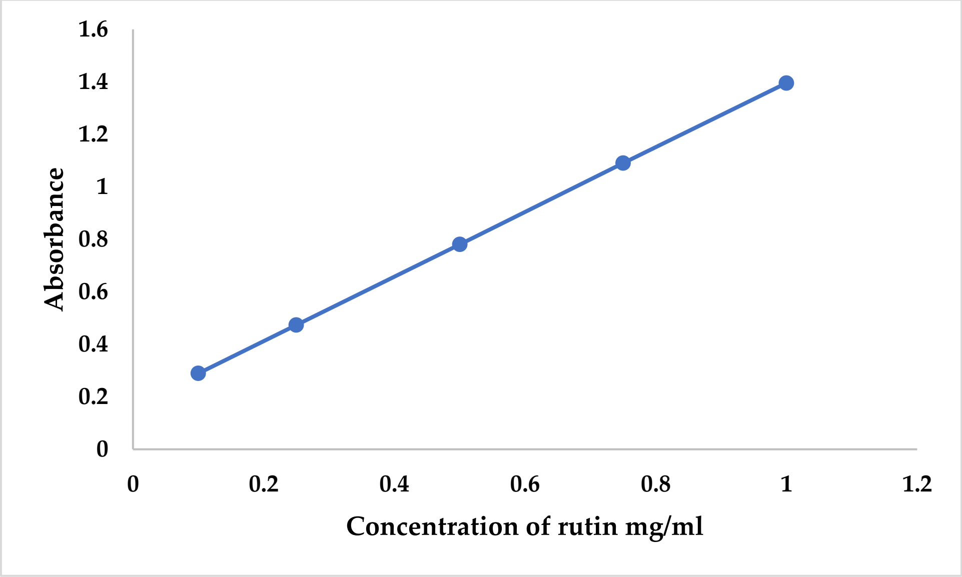


**Figure S5.** Calibration curve for rutin in the spectrophotometric assay.

**Table S1.** ANOVA Tukey HSD pairwise comparisons for the effect of raw material type on TPC.

| Comparison | Mean difference | Lower CI | Upper CI | p-value | Significant |
|---|---|---|---|---|---|
| Dried vs Freeze-dried | +3.11 | 1.16 | 5.06 | 0.0006 | Yes |
| Dried vs Frozen | +2.18 | 0.24 | 4.13 | 0.0238 | Yes |
| Freeze-dried vs Frozen | –0.93 | –2.87 | 1.02 | 0.498 | No |

**Table S2.** ANOVA Tukey HSD pairwise comparisons for the effect of ethanol concentration on TPC.

| Comparison | Mean difference | Lower CI | Upper CI | p-value | Significant |
|---|---|---|---|---|---|
| 30% vs 50% | +7.04 | 5.50 | 8.57 | <0.001 | Yes |
| 30% vs 70% | +2.42 | 0.89 | 3.95 | 0.0008 | Yes |
| 50% vs 70% | –4.62 | –6.15 | –3.09 | <0.001 | Yes |

**Table S3.** ANOVA Tukey HSD pairwise comparisons for the effect of temperature on TPC.

| Comparison | Mean difference | Lower CI | Upper CI | p-value | Significant |
|---|---|---|---|---|---|
| 20–25°C vs 50–55°C | –0.31 | –1.69 | 1.07 | 0.660 | No |

**Table S4.** ANOVA Tukey HSD pairwise comparisons for the effect of extraction time on TPC.

| Comparison | Mean difference | Lower CI | Upper CI | p-value | Significant |
|---|---|---|---|---|---|
| 10 min vs 30 min | –0.38 | –2.42 | 1.65 | 0.896 | No |
| 10 min vs 40 min | –0.17 | –2.21 | 1.86 | 0.978 | No |
| 30 min vs 40 min | +0.21 | –1.83 | 2.24 | 0.968 | No |

**Table S5.** ANOVA Tukey HSD pairwise comparisons for the effect of raw material type on TFC.

| Comparison | Mean diff | Lower CI | Upper CI | p-value | Significant |
|---|---|---|---|---|---|
| Dried vs Freeze-dried | +3.38 | 2.68 | 4.08 | <0.001 | Yes |
| Dried vs Frozen | +2.58 | 1.88 | 3.28 | <0.001 | Yes |
| Freeze-dried vs Frozen | –0.80 | –1.50 | –0.10 | 0.020 | Yes |

**Table S6.** ANOVA Tukey HSD pairwise comparisons for the effect of ethanol concentration on TFC.

| Comparison | Mean difference | Lower CI | Upper CI | p-value | Significant |
|---|---|---|---|---|---|
| 30% vs 50% | +1.60 | 0.69 | 2.51 | 0.0002 | Yes |
| 30% vs 70% | +1.16 | 0.24 | 2.07 | 0.0087 | Yes |
| 50% vs 70% | –0.44 | –1.36 | 0.47 | 0.486 | No |

**Table S7.** ANOVA Tukey HSD pairwise comparisons for the effect of temperature on TFC.

| Comparison | Mean diff | Lower CI | Upper CI | p-value | Significant |
|---|---|---|---|---|---|
| 20–25 °C vs 50–55 °C | –0.42 | –1.07 | 0.23 | 0.206 | No |

**Table S8.** ANOVA Tukey HSD pairwise comparisons for the effect of extraction time on TFC.

| Comparison | Mean diff | Lower CI | Upper CI | p-value | Significant |
|---|---|---|---|---|---|
| 10 vs 30 min | +1.00 | 0.06 | 1.95 | 0.034 | Yes |
| 10 vs 40 min | +0.73 | –0.21 | 1.67 | 0.163 | No |
| 30 vs 40 min | –0.27 | –1.22 | 0.67 | 0.771 | No |

**Table S9.** ANOVA Tukey HSD pairwise comparisons for the effect of raw material type on antioxidative activity by ABTS method.

| Comparison | Mean diff | Lower CI | Upper CI | p-value | Significant |
|---|---|---|---|---|---|
| Dried vs Freeze-dried | +26.17 | 5.29 | 47.05 | 0.0097 | Yes |
| Dried vs Frozen | +19.35 | –1.53 | 40.23 | 0.0756 | No |
| Freeze-dried vs Frozen | –6.83 | –27.71 | 14.05 | 0.7198 | No |

**Table S10.** ANOVA Tukey HSD pairwise comparisons for the effect of ethanol concentration on antioxidative activity by ABTS method.

| Comparison | Mean difference | Lower CI | Upper CI | p-value | Significant |
|---|---|---|---|---|---|
| 30% vs 50% | –35.99 | –56.38 | –15.60 | 0.0001 | Yes |
| 30% vs 70% | –21.49 | –41.88 | –1.10 | 0.0362 | Yes |
| 50% vs 70% | +14.49 | –5.90 | 34.88 | 0.2154 | No |

**Table S11.** ANOVA Tukey HSD pairwise comparisons of the effect of temperature on antioxidative activity by ABTS method.

| Comparison | Mean diff | Lower CI | Upper CI | p-value | Significant |
|---|---|---|---|---|---|
| 20–25 °C vs 50–55 °C | +17.59 | 3.25 | 31.93 | 0.0166 | Yes |

**Table S12.** ANOVA Tukey HSD pairwise comparisons for the effect of time on antioxidative activity by ABTS method.

| Comparison | Mean diff | Lower CI | Upper CI | p-value | Significant |
|---|---|---|---|---|---|
| 10 min vs 30 min | +4.33 | –17.11 | 25.78 | 0.8818 | No |
| 10 min vs 40 min | +7.33 | –14.11 | 28.78 | 0.6980 | No |
| 30 min vs 40 min | +3.00 | –18.45 | 24.45 | 0.9414 | No |

**Table S13.** ANOVA Tukey HSD pairwise comparisons for the effect of raw material type on antioxidative activity by DPPH method.

| Comparison | Mean diff | Lower CI | Upper CI | p-value | Significant |
|---|---|---|---|---|---|
| Dried vs Freeze-dried | +20.80 | 10.21 | 31.39 | <0.001 | Yes |
| Dried vs Frozen | –3.15 | –13.74 | 7.44 | 0.762 | No |
| Freeze-dried vs Frozen | –23.95 | –34.54 | –13.36 | <0.001 | Yes |

**Table S14.** ANOVA Tukey HSD pairwise comparisons for the effect of ethanol concentration on antioxidative activity by DPPH method.

| Comparison | Mean difference | Lower CI | Upper CI | p-value | Significant |
|---|---|---|---|---|---|
| 30% vs 50% | +3.76 | –7.14 | 14.66 | 0.6937 | No |
| 30% vs 70% | +20.71 | 9.81 | 31.61 | <0.001 | Yes |
| 50% vs 70% | +16.95 | 6.04 | 27.85 | 0.0009 | Yes |

**Table S15.** ANOVA Tukey HSD pairwise comparisons of the effect of temperature on antioxidative activity by DPPH method.

| Comparison | Mean diff | Lower CI | Upper CI | p-value | Significant |
|---|---|---|---|---|---|
| 20–25 °C vs 50–55 °C | –8.68 | –16.49 | –0.88 | 0.0295 | Yes |

**Table S16.** ANOVA Tukey HSD pairwise comparisons for the effect of time on antioxidative activity by DPPH method.

| Comparison | Mean diff | Lower CI | Upper CI | p-value | Significant |
|---|---|---|---|---|---|
| 10 vs 30 min | +0.75 | –10.88 | 12.38 | 0.987 | No |
| 10 vs 40 min | –3.66 | –15.29 | 7.97 | 0.738 | No |
| 30 vs 40 min | –4.41 | –16.04 | 7.22 | 0.643 | No |

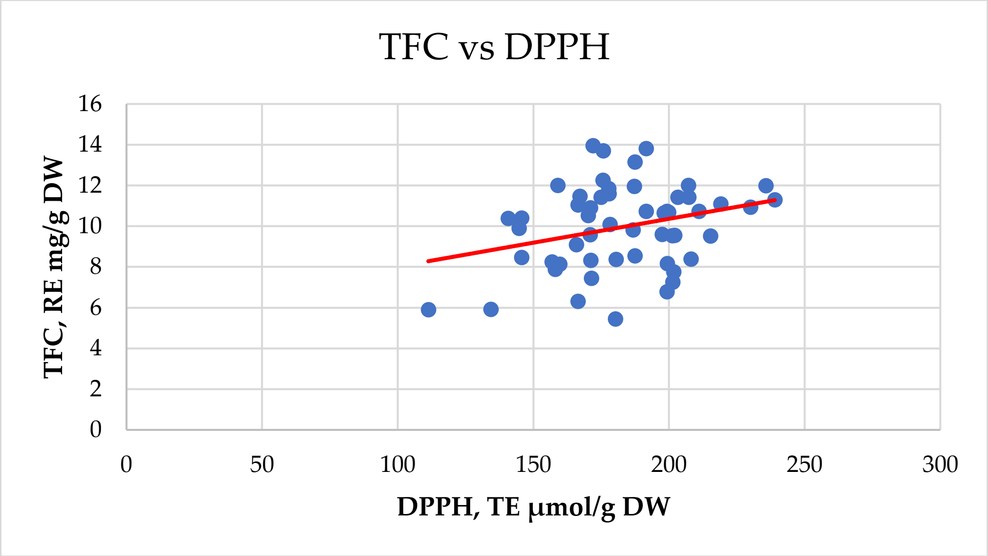


**Figure S6.** Correlation Between Total Flavonoid Content (TFC) and DPPH Radical-Scavenging Activity; r=0.293 (weak positive correlation), p=0.03.

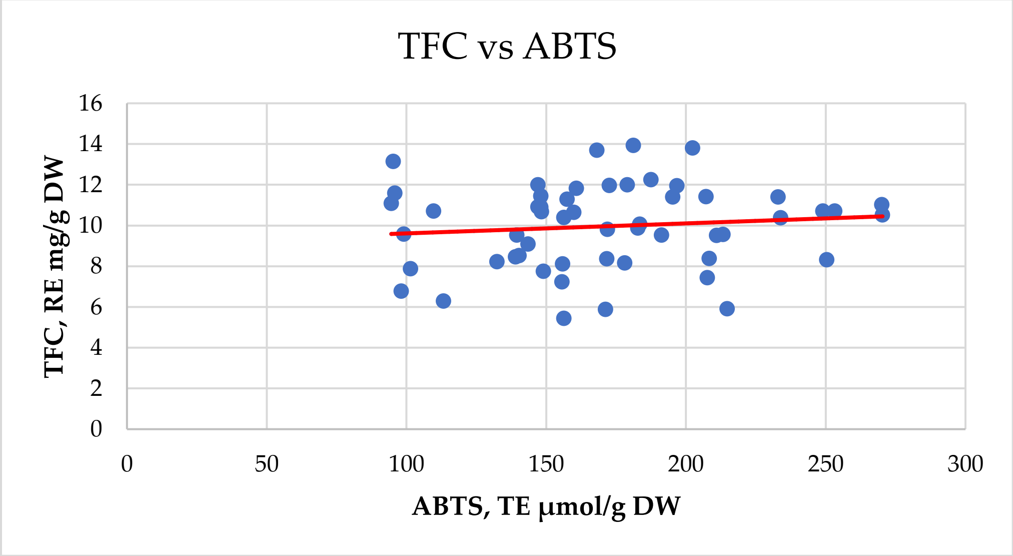


**Figure S7.** Correlation Between Total Flavonoid Content (TFC) and ABTS Radical-Scavenging Activity; r=0.108 (weak positive correlation), p=0.462.

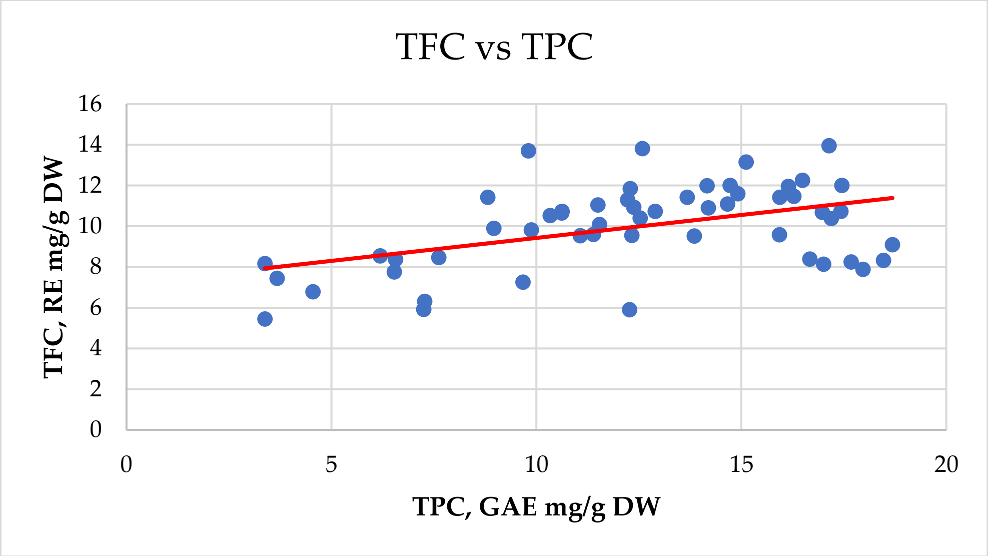


**Figure S8.** Correlation Between Total Flavonoid Content (TFC) and Total Phenolic Content (TPC; r=0.4625) (moderate positive correlation), p=0.0004.
